# Expression of microRNA-379 reduces metastatic spread of prostate cancer

**DOI:** 10.3389/fonc.2023.1252915

**Published:** 2023-09-12

**Authors:** James R. Cassidy, Gjendine Voss, Kira Rosenkilde Underbjerg, Margareta Persson, Yvonne Ceder

**Affiliations:** Department of Laboratory Medicine, Division of Translational Cancer Research, Lund University, Lund, Sweden

**Keywords:** microRNAs, prostatic cancer, neoplasm metastasis, disease management, tumour microenvironment, MIRN379

## Abstract

**Introduction:**

Prostate cancer (PCa) is the most common type of cancer in males, and the metastatic form is a leading cause of death worldwide. There are currently no curative treatments for this subset of patients. To decrease the mortality of this disease, greater focus must be placed on developing therapeutics to reduce metastatic spread. We focus on dissemination to the bone since this is both the most common site of metastatic spread and associated with extreme pain and discomfort for patients. Our strategy is to exploit microRNAs (miRNAs) to disrupt the spread of primary PCa to the bone.

**Methods:**

PCa cell lines were transduced to overexpress microRNA-379 (miR-379). These transduced PCa cells were assessed using cell growth, migration, colony formation and adhesion assays. We also performed *in vivo* intracardiac injections to look at metastatic spread in NSG mice. A cytokine array was also performed to identify targets of miR-379 that may drive metastatic spread.

**Results:**

PCa cells with increased levels of miR-379 showed a significant decrease in proliferation, migration, colony formation, and adhesion to bone cells *in vitro*. *In vivo* miR-379 overexpression in PC3 cells significantly decreased metastatic spread to bone and reduced levels of miR-379 were seen in patients with metastases. We identified GDF-15 to be secreted from osteoblasts when grown in conditioned media from PCa cells with reduced miR-379 levels.

**Discussion:**

Taken together, our *in vitro* and *in vivo* functional assays support a role for miR-379 as a tumour suppressor. A potential mechanism is unravelled whereby miR-379 deregulation in PCa cells affects the secretion of GDF-15 from osteoblasts which in turn facilitates the metastatic establishment in bone. Our findings support the potential role of miR-379 as a therapeutic solution for prostate cancer.

## Introduction

PCa is a leading cause of death worldwide and accounts for 29% of predicted cancer diagnoses in American men in 2023 (1). The majority of these individuals have the indolent form of the disease and can lead a normal lifestyle without presenting any symptoms at all (2). However, despite many advances in the field, patients with the metastatic form of the disease have a relative 5-year survival of only 28% compared to nearly 100% for the localised form (3), and there is still no curative treatment available for these individuals. This large discrepancy is a clear indication that research efforts in the field of PCa need to specifically address the manifestation of metastatic PCa. The most common site for metastatic spread is the bone, accounting for 70% of metastatic PCa patients (4), and it is also characterised by extreme pain and discomfort for the patients. The bone microenvironment is therefore the predominant focus of our research, as this represents the largest subset of patients with a poor prognosis. The cells that make up the bone are osteoblasts, osteoclasts, and osteocytes. Osteoblasts cause ossification, osteoclasts break down bone for remodelling, and osteocytes regulate biomineralisation (5). PCa bone metastases are typically osteoblastic, with unstable ossification leading to a fragile structure that is susceptible to fracturing. PCa is distinct from most bone metastases derived from other solid tumours, which are typically osteolytic in nature (6).

One potential avenue for developing novel therapeutics is using microRNAs (miRNAs). These non-coding single stranded RNA molecules are evolutionarily conserved and play a key role in post-transcriptional gene regulation. Typically, miRNAs bind to the 3’-untranslated region of a mRNA target, blocking its translation. Given the fundamental function of miRNAs, it is unsurprising that aberrant expression of these molecules has been implicated in many diseases, including PCa. Since miRNAs were initially proposed in 2002 to play a role in human cancer, there has been excitement for their potential clinical benefits (7). One key feature of miRNAs is their ability to regulate cellular plasticity (8). Cellular plasticity is a prerequisite for the metastatic development as it requires many dynamic shifts in cells to allow for invasion, intravasation, and extravasation into a distant site. By altering the levels of miRNAs through mimics or inhibitors, we may be able to reverse or halt the effects of PCa dissemination.

miR-379 is part of one of the largest evolutionarily conserved miRNA clusters which is located on chromosome 14q32. This region is often referred to as the *DLK1-DIO3* imprinted region, with miR-379 specifically residing between *MEG8* and *DIO3* (9). The miRNAs in this cluster are regulated by hypermethylation and MEF2. In addition, there are also reports of post-transcriptional RNA editing affecting the levels of the individual members of the miR-379 family (10–12). Specifically, we have shown that miR-379 editing is elevated in PCa and associated with decreased miR-379 levels, possibly a mechanism decoupling the miR-379 levels from the rest of the cluster (13). We have also shown that decreased expression of miR-379 is associated with decreased overall survival in patients and that patients with metastases had reduced expression of miR-379 in their primary tumours (13). In line with this, reduced levels of miR-379 were identified to be driving metastatic prostatic dissemination to bone (14). A tumour suppressor role has also been attributed to miR-379 in head and neck cancers, lung cancer, breast cancer, cervical cancer, gastric cancer, multiple myeloma, hepatocellular carcinoma, and osteosarcoma (15).

To investigate the therapeutic potential of miR-379 for patients with metastatic PCa, we adopted several different *in vitro* and *in vivo* experiments. We also aimed to explore the reciprocal signalling between PCa cells and the bone environment and determine how miR-379 dysregulation can impact this metastatic niche.

## Materials and methods

### Patient cohort

Relative miR-379 expression in benign tissue, primary tumour and bone metastasis was determined using the publicly available Taylor dataset set (16).

### Cell culture and transduction

Prostate cancer cell lines PC3 and LNCaP-ARhi and the osteosarcoma cell line, MG-63 were obtained from ATCC (American Type Culture Collection, Manassas, USA) and these were cultured as recommended by the supplier. These cell lines were validated and regularly tested for mycoplasma through Eurofins (eurofins scientific, Ebersberg, Germany). PC3 and LNCaP-ARhi cells were transduced with a commercially available Dharmacon shMIMIC Lentiviral miR-379 and Scr vector (GE Healthcare, CO) made using manufacturer’s protocol for our overexpressing cell line. These were selected with puromycin at a concentration of 1 µg/ml for both cell lines. 22Rv1 cell clones with the specific miRZip-379 microRNA constructs were used from the miRZip™ Lentivirus Pool and expanded (14) and used for the miR-379-inhibited cell lines. Luciferase transduction of miR-379 and Scr PC3 cells was performed by first co-transfecting HEK293T cells with a mixture of ENV plasmid (pMD2G_VSV-G), PsPax2 and a pLenti-CMV-V5-Luc-BlastR vector made in house. 30 h after transfection of HEK293T cells, the virus was collected from the media by centrifugation at 2500 rpm for 10 min and filtered through a 0.45 µm filter. Polybrene was added to this filtered virus before transducing seeded recipient cells. Antibiotic selection with blasticidin at a concentration of 4 µg/ml was started after 2 days of transduction.

### Acquiring osteoblast-conditioned medium and miR-379 conditioned media

Human primary mesenchymal bone marrow stem cells were a gift from Professor Stefan Scheding at the Lund Stem Cell Centre. These stem cells were cultured using StemMACS expansion medium (Miltenyi, Bergisch-Gladbach, Germany) and differentiated into osteoblasts using low glucose DMEM with 10% FBS with the addition of 10 mM β-glycerophosphate, 0.05 mM l-ascorbic acid, and 0.1 μM dexa-methasone (Sigma–Aldrich, Steinheim, Germany). Differentiated osteoblast cells were verified by staining with 10 mg/ml Alizarin Red (Sigma–Aldrich) after 3 weeks. Osteoblast-conditioned medium was collected after 48 h or 72 h, and cell debris was cleared by centrifugation at 900g. For experiments in osteoblast-conditioned medium, a 1:1 ratio was used with the cell lines normal media. For miR-379 conditioned media, PC3 cells transduced with miR-379 were grown in normal media, which was collected after 48 h or 72 h and cell debris cleared by centrifugation at 900g. The same method was used for anti-miR-379 media; however, the 22Rv1 cells transduced with anti-miR-379 were used. If the media was not used immediately for an experiment, it was stored at -80°C.

### RT-qPCR to measure miR-379 levels

Samples were reverse transcribed with the qScript Flex Kit (#95049-100, Quantabio) using 2 μL 5× reaction mix, 1 μL GSP enhancer, 0.05 μM two-tailed RT primer, and 0.5 μL reverse transcriptase in a total volume of 10 μL. RT was performed at 25°C for 1 h, stopped at 85°C for 5 min, and samples were held at 4°C. The input for RT was 2 μg RNA. The qPCR was performed with PowerUp SYBR Green Master Mix (#A25742, Thermo Scientific) using 400 nM forward and reverse primers as described by Voss et al. (7, 13) Samples were assayed in triplicates using the QuantStudio 7 Flex qPCR machine (Applied Biosystems). The qPCR programme consisted of 30 sec of initial denaturation at 95°C, followed by 45 cycles of 5 sec at 95°C and 20 sec at 60°C. Melt curve analysis was performed to exclude the amplification of unspecific products. TaqMan microRNA assays (Applied Biosystems, Pleasanton, CA) were used for small RNA housekeeping controls according to the manufacturer’s instructions with 100 ng RNA input. Then miR-379 was normalised to the geometric mean of RNU44 (#001094) and RNU48 (#001006) using the ΔCt method. We did not measure the levels of miR-379 inhibition by RT-qPCR since the miRZip-379 microRNA construct transiently binds to miR-379, and miR-379 is therefore not expected to be reduced.

### Growth assay

The sulforhodamine B assay was used to assess cell growth. 20,000 cells were seeded in 6-well plates and cultured for 16 h (normalisation), 24 h, 48 h, 72 h, and 96 h, and then fixed in 10% trichloroacetic acid. These were subsequently stained with 0.4% sulforhodamine B (Sigma–Aldrich, MO) in 1% acetic acid for 15 min. Unbound sulforhodamine B was washed away with tap water, and the remaining sulforhodamine B was dissolved in 10mM Tris base. Absorbance was read at 690nm to determine cell density using the Synergy 2 plate reader (BioTek, VT), and normalised to the 16 h attachment control.

### Migration assay

The Boyden chamber method was used to assess cell migration in 6-well plates. Cells were trypsinised and made up to a concentration of 2.1x10^5^ cells/ml in serum-free media. This was added to the inside of the polycarbonate membrane with 8µm pores, Catalogue number 3428, (Corning, ME) and 2 ml serum-positive medium (with and without OBCM) was added to the well outside of the insert. Wells with serum-free media on the outside of the insert were used for normalisation. The cells were then fixed after 16 h with 100% methanol and stained with 0.1% Crystal Violet solution in 20% methanol (Sigma-Aldrich). Unbound Crystal Violet was removed by washing with tap water and swabbing the inner chamber of the insert with cotton buds. The migrated cells were then detached in 1.2 ml 10% of acetic acid. Triplicates of 100 µl were transferred onto a 96-well plate, and absorbance was read at 595 nm using a Synergy 2 plate reader (BioTek, VT).

### Colony formation assay

Colony formation assay was performed in 6-well plates. The bottom layer was made up of 0.5% agarose (Saveen & Werner, Sweden) in 2 mL of appropriate medium. 1,000 cells were seeded in each well containing 0.3% agarose in normal growth medium or growth medium mixed 1:1 with osteoblast-conditioned medium. After the agarose set, 1 ml of the respective growth medium was added to prevent drying of the agarose. After colonies were visibly present, they were fixed in 100% methanol and, after 1 hr, stained with 0.01% crystal violet (Sigma-Aldrich) in 20% methanol for 15 mins. Water was used to remove the background colour until colonies were clearly visible.

### Adhesion assay

100,000 of the cells of interest were seeded in 12-well plates to create a monolayer. The following day, the prostate cancer cells were detached with Versene (Thermo-Scientific) and resuspended in PBS. These were then spun down at 300 xg for 5 min and then resuspended in 1 ml of BCECF diluted 1:400 in PBS (#B3051, Fisher Scientific, OR) and incubated at 37°C for 15 min. Cells were resuspended in media to get a final concentration of 8×10^4^ cells/ml. 500 µl of the cell suspension was added on top of the monolayer of cells and incubated at 37°C for 3 h. Cell media containing non-adherent cells were removed, and the remaining adhered cells were lysed with 100 µl 5x passive lysis buffer (Promega, WI). These were incubated for 15 min on an orbital shaker before 45 µl duplicates were transferred to a 96-well plate. Fluorescence was measured (excitation 490 nm, emission 535 nm).

### *In vivo* intracardiac injection

Cells were trypsinised and made up in ice-cold antibiotic-free medium. The cell suspension was then centrifuged at 200 xg. The pellet was resuspended in ice-cold PBS and centrifuged again at 200 xg. The cells were then counted and made up to a concentration of 5x10^6^ cells/ml. Mice were anaesthetised using IsoFlo® vet isoflurorane (Apotek Hjärtat AB, Sweden) at a concentration of 4% with an airflow of 300 ml/min using the Univentor 410 Anaesthesia unit (Agntho’s AB, Sweden). After the mice were unconscious, the chest was shaved, and 100 ul of the cell suspension was injected intracardially using a 30G, 0.5ml Insulin needle (Becton-Dickinson, NJ). In total, 24 mice were injected, but two of the mice were excluded later due to the development of tumours in the heart, which may indicate that cells were injected into the heart wall as opposed to the blood stream. The experiment was conducted according to the guidelines from the regional Ethics Committee for Animal Research, permit number 11883_19. Imaging of the mice was done after they were injected subcutaneously with D-luciferin (150 mg/kg, PerkinElmer, MA) in PBS using the IVIS Spectrum *In Vivo* Imaging System (PerkinElmer, MA). Mice were placed inside the imaging chamber under continuous exposure to isoflurane. In total, 4 images were taken with 5 min intervals between them. The most suitable image was then analysed with Living Image 4.5.5 software (PerkinElmer). Bioluminesence image signal intensity was quantified in total flux (photons/s) after deducting the average background signal from the measurement region of interest (ROI) around the tumour area using live image analysis software (PerkinElmer, MA). Bones from mice were taken after imaging and fixed in 4% formalin. After 48 h bones were moved to 10% EDTA, pH 7.5, for 96 hours before returning to 4% formalin until they were dehydrated and embedded in paraffin. Tissue sections were cut into 4 μm sections. H&E (Histolab Products, Sweden) staining was performed for histopathological analysis. Representative images were taken at 40x magnification, and the slides were blinded for morphological assessment and evaluated by an independent viewer.

### Cytokine array

Osteoblasts that had been differentiated in 6-well plates from human primary mesenchymal bone marrow stem cells as described above were used for a cytokine array. Conditioned media from 22Rv1 anti-miR-379 cells and 22Rv1 Scr cells were individually mixed with OBCM at a ratio of 1:1. This media was added to the osteoblast cells for 72 hours before being replaced with fresh osteoblast media for a further 18 hours, after which the supernatant was collected. The array was that of a commercial kit; Proteome Profiler Human XL Cytokine Array Kit (BioTek, VT). The protocol was followed as according to manufacturer’s instructions, with the initial incubation step being left overnight at 4°C and imaging exposure run for 30 sec. A cut-off was set at 60 QL/pixel^2^ and any cytokines below this threshold were excluded.

### GDF15 ELISA and qPCR

The Solid Phase Sandwich ELISA used to measure GDF15 expression was the Human GDF-15 Quantikine ELISA Kit #DGD150 kit (Bio-Techne, MN). The protocol was followed according to the manufacturer’s instructions. In brief, the conditioned media was added to 96-well plates before a conjugate was added, and then a substrate solution. After 30 mins in the darkness, stop solution was added and the plate was read using a Synergy 2 plate reader at 450 nm (BioTek, VT). Total RNA from PNT2 and PCa cell lines was treated with DNase I (Thermo Scientific, Lithuania) according to the manufacturer’s instructions at 37°C for 30 min. Total cDNA from 3 μg of RNA was synthesised using the RevertAid H Minus First Strand cDNA Synthesis kit (#K1632, Thermo Scientific) according to the manufacturer’s instructions. The cDNA was diluted 1:5, and 1 μl of the diluted cDNA was used for qPCR in triplicates. TaqMan Gene expression assays and TaqMan Gene expression Master Mix (#4369016, Thermo Scientific) were used according to the manufacturer’s instructions on a QuantStudio 7 Flex machine. Expression of *GDF-15* (Hs00171132_m1) mRNA was normalised to the geometric mean of *GUSB* (Hs00939627_m1) and *GAPDH* (Hs02758991_g1) mRNAs using the ΔCt method.

### Statistical analysis

Statistical analysis was done using GraphPad Prism 9 (GraphPad software, La Jolla, CA) and statistical significance was determined using either a two-tailed unpaired Student’s t-test, Mann-Whitney or Chi-square test. A Shapiro-Wilk test was used to confirm normal distribution in the groups. A p value of < 0.05 was considered statistically significant.

## Results

### miR-379 expression in PCa patients and cell lines

**Figure 1:**
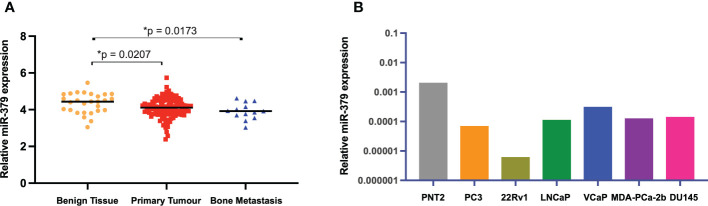
miR-379 expression levels in patients and cell lines. The relative levels of miR-379 are shown **(A)** in an external patient cohort consisting of miRNA transcript levels of benign tissue from 28 men without PCa (yellow circles), 122 men with PCa (red squares), and 19 men with bone metastasis (blue triangles). The bar chart **(B)** shows baseline expression of several prostate cancer cell lines and an immortalised normal prostate cell line (PNT2, grey). Unpaired two-tailed Student’s *t*-tests were performed to compare the treatment groups to one another; *p < 0.05. A Shapiro–Wilk test was used to confirm normal distribution in the groups. Only statistically significant *p* values are shown in the figure.

We started off by examining the levels of miR-379 in an external cohort of patients with benign hyperplasia, primary tumours, and metastasis samples. The levels of miR-379 were found to be higher in benign prostatic tissue compared to patients with primary prostate cancer or bone metastases but there was no difference in miR-379 levels between primary tumour and bone metastasis (**Figure 1A**). We then used RT-qPCR to measure miR-379 levels in various prostate cell lines. PCa cell lines were found to have relatively low expression of miR-379 compared to an immortalised normal prostate tissue cell line, PNT2 (**Figure 1B**) in line with what was found in the patient cohort. PC3 and LNCaP-ARhi cells were transduced with miR-379 and a Scrambled control (Scr), and miR-379 levels were measured using RT-qPCR. Highly increased expression of miR-379 was found in the transduced cell lines, confirming that the transduction was successful (**Supplementary Figures 1A, B**). Then both PC3 379 and PC3 Scr cell lines were successfully transduced with a luciferase vector, as shown using the IVIS imaging of a cell line serial dilution in the 96-well plate (**Supplementary Figure 1C**).

### Effect of miR-379 overexpression on cell growth, migration, and colony formation

**Figure 2:**
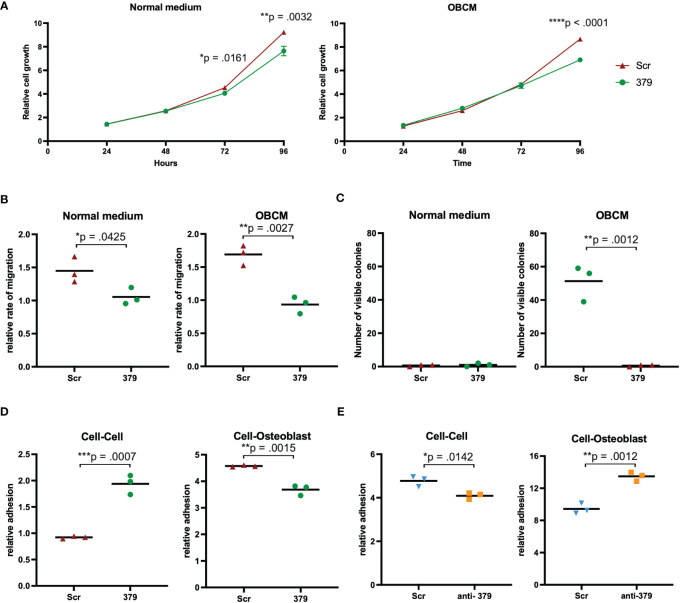
Effect of miR-379 overexpression *in vitro.* PC3 cells transduced with miR-379 were assessed for the effect of miR-379 on cell growth **(A)**, migration **(B)**, colony formation **(C)**, and cellular adhesion **(D, E)**. For the SRB **(A)**, migration **(B)**, colony formation **(C)**, and cellular adhesion assays **(D)**, the red triangles denote Scr control cells, and the green circles denote miR-379 overexpressed PC3 cells. For the cellular adhesion assays the blue triangles denote Scr control cells, and the orange boxes denote anti-miR-379 22Rv1 cells. The functional experiments **(A–C)** were performed in normal media and OBCM. Both adhesion experiments looked at cell-cell adhesion and cell-Osteoblast adhesion. Experiments were performed three times, and representative data is shown. Unpaired two-tailed Student’s *t*-tests were performed to compare the treatment groups to one another; *p < 0.05, **p < 0.01; ***p < 0.001; ****p < 0.0001. Only statistically significant *p* values are shown in the figure.

The effect of miR-379 overexpression was examined *in vitro* in normal growth medium and OBCM. This OBCM is used to mimic the bone microenvironment, given that PCa usually forms osteoblastic lesions in the bone (6). In transduced PC3 cells, miR-379 showed an inhibitory effect on cell growth over 96 hours, and this was more pronounced in the OBCM setting (**Figure 2A**). In the LNCaP-ARhi cells growth was also reduced with miR-379 overexpression in normal media and OBCM (**Supplementary Figure 2A**). There was also an inhibitory effect on migration in the transduced PC3 cells when miR-379 was overexpressed, which was more pronounced in the OBCM setting (**Figure 2B**). This was also the same general trend in LNCaP-ARhi cells (**Supplementary Figure 2B**). Colony formation was accelerated in the OBCM setting, where the overexpression of miR-379 inhibited the ability of cells to form colonies (**Figure 2C**, **Supplementary Figure 3**). No colony formation could be seen for LNCaP-ARhi cells.

### miR-379 overexpression increases cell-cell adhesion and decreases cell-bone adhesion

Adhesion assays were set up to look at adhesion between PCa cells as well as between PCa and osteoblasts or MG-63, an osteosarcoma cell line. When performing adhesion assays, there was significantly more cell-cell adhesion between PC3 cells with increased levels of miR-379 (**Figure 2D**), but significantly less adhesion between PC3 379 cells and osteoblast cells (**Figure 2D**) or MG-63 (**Supplementary Figure 4**). The opposite was seen when reducing the levels of miR-379 in 22Rv1 cells compared to Scr control: reduced cell-cell adhesion between the PCa cells (**Figure 2E**), but increased cell-osteoblast adhesion (**Figure 2E**) and cell-MG-63 adhesion (**Supplementary Figure 4**).

### miR-379 overexpression reduces metastatic spread *in vivo*

**Figure 3:**
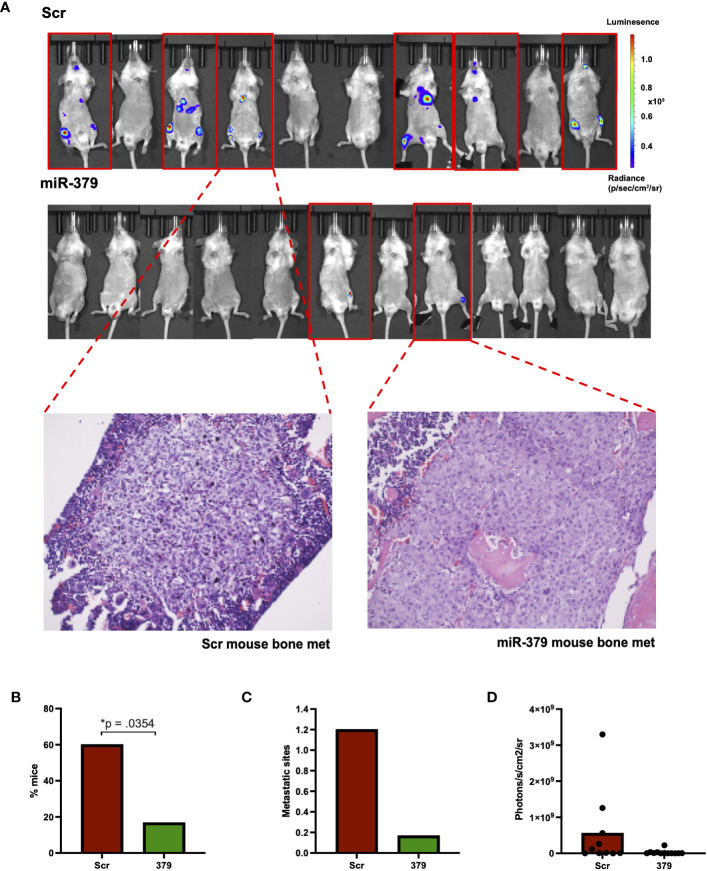
Effect of miR-379 overexpression *in vivo*. PC3 cells transduced with miR-379 and luciferase were injected into 22 NSG mice, with 6/10 mice in the scrambled condition developing metastasis and 2/12 mice in the mir-379 overexpressed condition developing metastasis, as shown in the red border **(A)**. Tissue sections taken from the legs of mice after hematoxylin and eosin staining under 20x magnification. These samples were taken from mice that showed a clear IVIS signal for bone metastases, as shown by the dotted red line. The percentage of mice with metastasis is shown **(B)** as well as the number of metastatic sites **(C)**. Average photons measured from individual mice are also shown **(D)**. A chi-square test was used for figure **(B)** and Mann-Whitney tests were used on figures **(C, D)** as data points were not normally distributed in the groups using the Shapiro–Wilk test; *p < 0.05. Only statistically significant *p* values are shown in the figure.

To investigate the effect of miR-379 on metastatic spread to the bone *in vivo*, we performed intracardiac injections, and of the 22 successfully injected mice, ten were injected with PC3 Scr cells and twelve were injected with PC3 379 cells. In total, six of the ten (60%) PC3 Scr-injected mice developed bone metastases, whereas only two of the twelve (17%) PC3 379-injected mice developed metastases in the bone (**Figures 3A, B**). Not only was there a stark reduction in metastatic spread to bone when miR-379 was overexpressed, but the number of metastatic sites within each mouse was significantly reduced, with most of the PC3 Scr mice obtaining multiple metastatic sites in different skeletal regions (**Figure 3C**). Based on the relative signal strength, there is also a trend for smaller tumours in the miR-379 overexpressed mice compared to the Scr control (**Figure 3D**). Bone tissue sections stained with H&E from the bone metastasis of PC3 Scr and PC3 379 mice also show a clear difference in morphology (**Figure 3A**). The metastases in the Scr mice were more heterogeneous and less differentiated than the bone metastases in the miR-379 overexpressing ones. The LNCaP-ARhi 379/Scr cell lines were also injected into mice; however, none of the seven mice developed any bone metastasis.

### miR-379 deregulation impacts GDF-15 expression in bone cells

**Figure 4:**
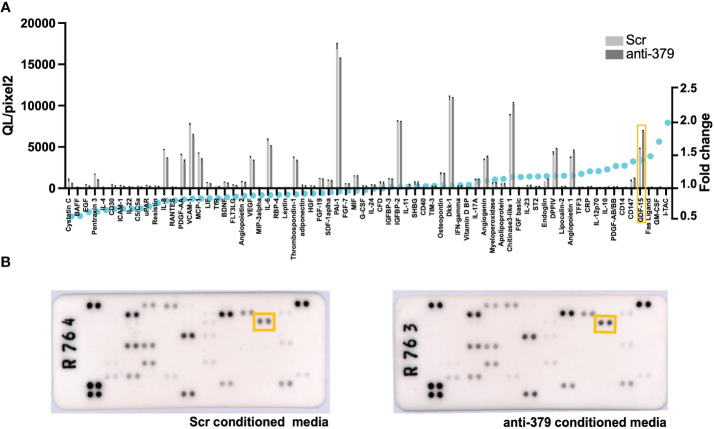
Cytokine array on osteoblast cells grown in anti-miR-379-conditioned media. The waterfall plot **(A)** shows the cytokine expression as measured in QL/Pixels^2^ in the left axis and fold change (displayed as blue circles) in the right axis of MG-63 cells grown in anti-miR-379 22Rv1 cell conditioned media compared to Scr 22Rv1 cell conditioned media. GDF15 is highlighted in the orange box. The raw blots **(B)** are also shown with the corresponding GDF15 highlighted within the orange boxes.

**Figure 5:**
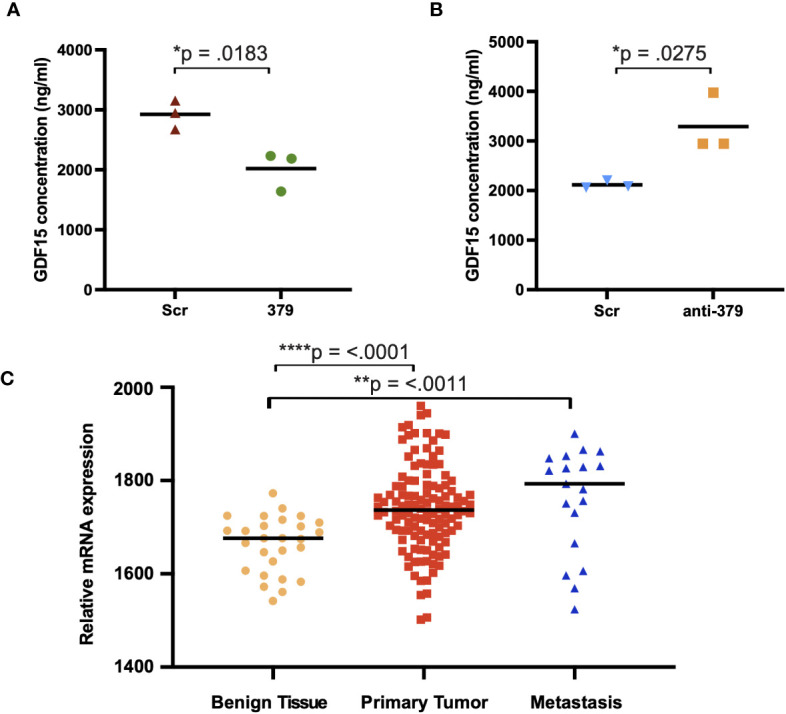
GDF15 expression in MG-63 cells grown in miR-379/anti-miR-379 conditioned media. Media from PC3 cells transduced with miR-379 **(A)** and 22Rv1 cells transduced with anti-miR-379 **(B)** were added to MG-63 cells before ELISA. The red/blue triangles denote Scr-control conditioned media, and the green circles/orange boxes denote miR-379 and anti-miR-379 conditioned media. Experiments were performed three times, and representative data is shown. Individual values of biological triplicates are shown as red or blue triangles, green circles, and orange squares. The relative levels of GFRAL are also shown **(C)** in an external patient cohort consisting of mRNA transcript levels of benign tissue from 28 men without PCa (yellow circles), 122 men with PCa (red squares), and 19 men with bone metastasis (blue triangles). Unpaired two-tailed Student’s *t*-tests were performed to compare the treatment groups to one another; *p < 0.05; **p < 0.01; ****p < 0.0001. A Shapiro–Wilk test was used to confirm normal distribution in the groups. Only statistically significant *p* values are shown in the figure.

To further investigate the mechanism behind the impact of miR-379 on PCa bone metastases establishment, osteoblasts were grown in conditioned media from anti-miR-379 transduced 22Rv1 cells or control 22RV1 cells and then a cytokine array was performed after the media change. Several secreted cytokines were identified to be increased in the conditioned media of osteoblasts grown in anti-miR-379-conditioned media compared to conditioned media from the Scr control, and GDF-15 was found to be highly induced and expressed at high levels (**Figure 4**). To validate if miR-379 levels in PCa cells could impact GDF-15 secretion by bone cells, we performed an ELISA using MG-63 cells grown in conditioned media from both miR-379-overexpressing PC3 cells and conditioned media from anti-miR-379-transduced 22Rv1 cells, with respective controls. We found a significant decrease in GDF15 secretion from MG-63 cells grown in PC3 379 conditioned media compared to PC3 Scr conditioned media (**Figure 5A**). We also found a significant increase in GDF15 secretion from MG-63 cells grown in 22Rv1 anti-miR-379 conditioned media compared to 22Rv1 Scr conditioned media (**Figure 5B**), in agreement with the results from the cytokine array. We also investigated an external patient cohort consisting of 28 normal prostate samples, 98 primary tumours, and 13 bone metastases (16) to investigate the levels of GFRAL, the receptor for GDF-15, in prostate tissues. The levels of GFRAL mRNA expression were significantly higher in metastases and primary tumours compared to the benign hyperplasia group (**Figure 5C**). When looking at GDF-15 expression in PCa cell lines, the PNT2 cell line has the lowest relative expression, alongside the brain-metastasis derived DU145 line (**Supplementary Figure 5**).

## Discussion

We have previously shown that decreased levels of miR-379 elicited bone metastases *in vivo* (14) and patients with metastases had lower miR-379 levels in their primary tumours, suggesting that the downregulation of miR-379 can drive the metastatic spread of PCa (13). In line with this, the present study shows that relative miR-379 expression was lower in primary tumour and bone metastasis compared to benign tissue and in all PCa cell lines compared to PNT2, the normal prostate tissue cell line. Intriguingly, the cell line with lowest overall expression was 22Rv1 a cell line of epithelial origin, which has been serially propagated in castrated mice (17) and hence mimics castration resistant PCa, *i.e.* late stages of PCa. This may be the reason for its low relative expression compared to the other metastasis-derived cell lines. The tumour suppressor role of miR-379 is also seen in other cancers, such as cervical and breast cancer (18, 19).

We have here shown promising *in vitro* results that many of the metrics associated with cancer progression, such as cell growth, migration, and colony formation, are diminished by miR-379. The reduction of cell growth is consistent with what has been seen previously in head and neck cancers as well as lung cancer (20–22). The effect of miR-379 to reduce migration has also been seen in other cancer types, notably laryngeal carcinoma, hepatocellular carcinoma, and nasopharyngeal cancer (20, 23, 24). The most striking phenotype of miR-379 is its ability to impede single cells from forming colonies in a bone-like environment, and this has previously been reported in nasopharyngeal cancer (24). In the present study, there was only a difference between miR-379 and Scr control cells in the OBCM setting and not the normal media. This is consistent with our previous findings that show decreased miR-379 levels specifically lead to dissemination to bone and increased colony formation in soft agar assays in OBCM only (14). This key metastatic process being interrupted is likely one of the main explanations for the effect seen in our *in vivo* findings.

From our *in vivo* experiments there was not only a stark difference in the metastatic spread of PC3 with miR-379 injected mice compared to Scr control but also some interesting findings about the nature of these metastases. Firstly, the miR-379 overexpressing cells spread mainly to the femurs while the Scr control cells had multiple additional metastatic sites such as the rib cage and mandible of the mouse that were not seen in miR-379 injected mice. Secondly, the physiology of the metastases in the mice injected with miR-379-overexpressing cells appeared more differentiated and less heterogeneous. In future studies it would be interesting to explore in depth the effect of miR-379 on the cellular composition of bone metastasis as it could give better insights about its biological role in PCa development.

The use of OBCM to mimic the bone environment *in vitro* is a limitation of the study. Although OBCM is a much better way of constituting a bone environment *in vitro* than normal conditioned media, it is still rudimentary as it encompasses signalling factors from only one cell type, whereas the bone is made up of three distinct cell populations: osteoblasts, osteoclasts, and osteocytes. Despite this, it is still interesting to note how OBCM affects the *in vitro* PC3 experiments, especially colony formation, and that this seems to be a representative model of what is happening *in vivo*. Another limitation of this study is the use of only one PCa cell line in our *in vivo* experiments. Despite several attempts to inject LNCaP-ARhi cell line in the mouse model, there was not any metastatic spread of cells to the bone.

The aim of this study was to investigate the therapeutic potential of miR-379 for reducing the metastatic spread of PCa to the bone. The bone is of particular interest given that it is the most common metastatic site for patients with PCa (4). We performed adhesion assays since the ability of cells to detach from other cancer cells at the primary site and instead adhere to bone cells at the metastatic site is essential in the metastatic process. The adhesion assays further support the notion of miR-379 as a metastatic suppressor in PCa, as increased levels of miR-379 increased cell-cell adhesion but decreased adhesion of PCa to osteoblasts. The opposite was seen when miR-379 levels were decreased; decreased cell-cell adhesion and increased cell-osteoblast adhesion. Adhesion of PCa cells to MG-63 cells followed the same pattern as cell-osteoblasts, and this is unsurprising given that osteosarcoma originates from transformed pre-osteoblasts (25). Taken together, these results clearly show the impact of miR-379 on adherence to cells that make up the bone niche. The promising findings *in vitro* have been supported by *in vivo* evidence that the spread of PCa cells to the bone is reduced with miR-379 overexpression. There is a previous report suggesting an oncogenic role of miR-379 in PCa (26). However, they do not show *in vivo* results for miR-379, and the *in vitro* data showing increased mesenchymal–epithelial transition upon miR-379 decrease is only performed on the ARCaPM cell line. This makes comparisons difficult, but indicates that further studies in more complex models are needed.

We wanted to unravel more about the biological pathways in which miR-379 is involved. Our cytokine array uncovered an interesting target expressed in the osteoblasts, GDF-15, a cytokine known to be involved in the establishment of bone metastasis (27–30). The relative expression of the GDF-15 receptor, GFRAL, was higher in metastases compared to benign hyperplasia. GDF-15 is involved in the stress response and is known to be overexpressed by most cancers (27). In addition to being expressed in the osteoblast, it is also expressed in the PCa cells, and all our PCa cell lines, apart from DU145, showed higher GDF-15 expression than the normal immortalised PNT2 cell line. It has also been reported that PC3 cells transduced with GDF-15 show increased proliferation, migration, colony formation, and metastatic spread *in vivo* (29). GDF-15 has been suggested to promote bone metastasis through a feedback loop between the bone niche and PCa cells, with GDF-15 secreted from bone cells promoting GDF-15 secretion from PCa cells and vice versa (29, 30). The exact role of GDF-15 in the bone microenvironment is not fully understood, but there is evidence that through the action of the CCL2 chemokine, GDF-15 can recruit osteal macrophages, which are important in maintaining the bone during stress (30). GDF-15 secreted from PCa cells can also increase osteoclast formation (30). Taken together, GDF-15 is important in bone remodelling during metastatic invasion of PCa cells. These results could suggest a role whereby the action of miR-379 on GDF-15 primes the bone microenvironment so that PCa metastases are more permissible in this niche.

The goal of this research was to determine if miR-379 could ultimately be used as a feasible therapeutic for PCa. Our data shows promise, and there have been similar findings in other cancer systems supporting this. Previous research has found that the use of miR-379 mimics can inhibit osteosarcoma progression *in vivo* (31). In this study established subcutaneous tumours were injected directly with miR‐379 mimics or a Scr control. Another method of miRNA delivery that has been discussed is extracellular vesicles, since these can stably deliver miRNAs using blood as a medium along short and long distances (32). There has also been a study focusing on using mesenchymal stem cells to home in on breast cancer cells and secrete extracellular vesicles enriched with miR-379, whereby a therapeutic effect was observed *in vivo* (33). The results of these studies show that not only can miR-379 effectively stunt cancer growth in model systems but successful delivery of this miRNA to cancer cells is possible. In conclusion we have demonstrated through both *in vitro* and *in vivo* data that miR-379 overexpression can reduce the metastatic spread of PCa to the bone. This is something that merits further investigation, as it has the potential to be of clinical relevance for many men suffering from the consequences of PCa bone metastases.

## Data availability statement

The original contributions presented in the study are included in the article/**Supplementary Material**. Further inquiries can be directed to the corresponding author.

## Ethics statement

Ethical approval was not required for the studies on humans in accordance with the local legislation and institutional requirements because only commercially available established cell lines were used. The animal study was approved by regional Ethics Committee for Animal Research, permit number 11883_19. The study was conducted in accordance with the local legislation and institutional requirements.

## Author contributions

JC: Experimental design, running of experiments, data analysis, figure design, writing of manuscript. GV: Running of experiments, writing of manuscript. KU: Running of experiments. MP: Running of experiments. YC: Conception and design of the study, securing funding, writing of manuscript. All authors contributed to the article and approved the submitted version.

## Supplementary material

Supplementary Figure 1 miR-379 and luciferase expression in transduced PC3 and LNCaP-ARhi cell lines. Bar chart to show fold change in miR-379 in PC3 **(A)** and LNCaP-ARhi **(B)** cells transduced with miR-379 (green bar) as opposed to those transduced with the Scr control (red bar). IVIS imaging of PC3 379 and Scr cells transduced with a luciferase vector seeded in a 96-well plate **(C)**. This serial dilution between columns 2 and 11 starts at 1.6 million cells seeded in row 2 and is decreased by half in each column until only 3000 cells are seeded in column 11. Rows B and C contain PC3 379/Luc cells, and rows E and F contain PC3 Scr/Luc cells. Images were taken 5 mins and 10 mins after being placed in the IVIS Spectrum *In Vivo* Imaging System respectively.

Supplementary Figure 2 Effect of miR-379 overexpression on LNCaP-ARhi cells *in vitro*. LNCaP-ARhi cells transduced with miR-379 were assessed for the effect of miR-379 on cell growth **(A)** and migration **(B)**. For the SRB assays **(A)**, the red boxes denote Scr control cells, and the green circles denote miR-379 overexpressed cells. For the migration **(B)** experiments, the red triangles denote Scr control cells, and the green circles denote miR-379 overexpressed cells. The experiments were performed in normal media and OBCM. Experiments were performed three times, and representative data is shown. Unpaired two-tailed Student’s *t*-tests were performed to compare the treatment groups to one another; **p* < 0.05. Only statistically significant *p* values are shown in the figure.

Supplementary Figure 3 Plates from the colony formation experiment. Scanned images of the 6-well plates from which the number of visible colonies was counted. The number of colonies can be seen in .

Supplementary Figure 4 Effect of miR-379 overexpression and downregulation on cellular adhesion to MG-63 cells. The red/blue triangles denote Scr control cells, and the green circles/orange boxes denote miR-379 and anti-miR-379 cells. Experiments were performed three times, and representative data is shown. Individual values of biological triplicates are shown as blue triangles and orange squares. Unpaired two-tailed Student’s *t*-tests were performed to compare the treatment groups to one another; **p* < 0.05, ****p* < 0.001. Only statistically significant *p* values are shown in the figure.

Supplementary Figure 5 GDF-15 expression levels in cell lines. The bar chart shows baseline expression of several prostate cancer cell lines and an immortalised normal prostate cell line (PNT2, grey).
