# Supplementary figures and images for: Expression of microRNA-379 reduces metastatic spread of prostate cancer
Source: Front Oncol. 2023 Sep 12;13:1252915. doi: 10.3389/fonc.2023.1252915 (PMC10539900; doi:10.3389/fonc.2023.1252915)

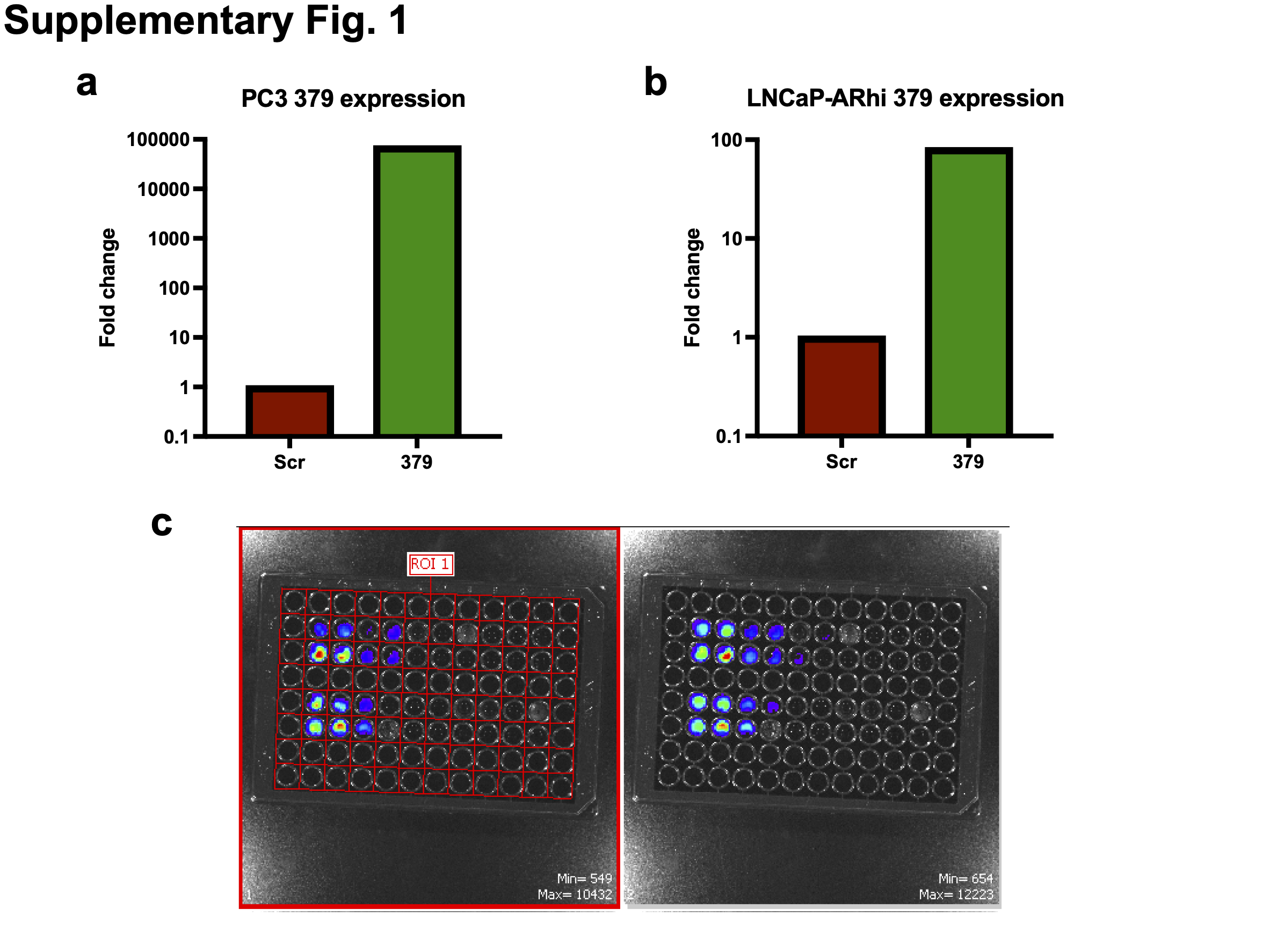

Supplement: Supplementary Figure 1 — miR-379 and luciferase expression in transduced PC3 and LNCaP-ARhi cell lines. Bar chart to show fold change in miR-379 in PC3 (A) and LNCaP-ARhi (B) cells transduced with miR-379 (green bar) as opposed to those transduced with the Scr control (red bar). IVIS imaging of PC3 379 and Scr cells transduced with a luciferase vector seeded in a 96-well plate (C). This serial dilution between columns 2 and 11 starts at 1.6 million cells seeded in row 2 and is decreased by half in each column until only 3000 cells are seeded in column 11. Rows B and C contain PC3 379/Luc cells, and rows E and F contain PC3 Scr/Luc cells. Images were taken 5 mins and 10 mins after being placed in the IVIS Spectrum In Vivo Imaging System respectively. [file Image_1.tiff]

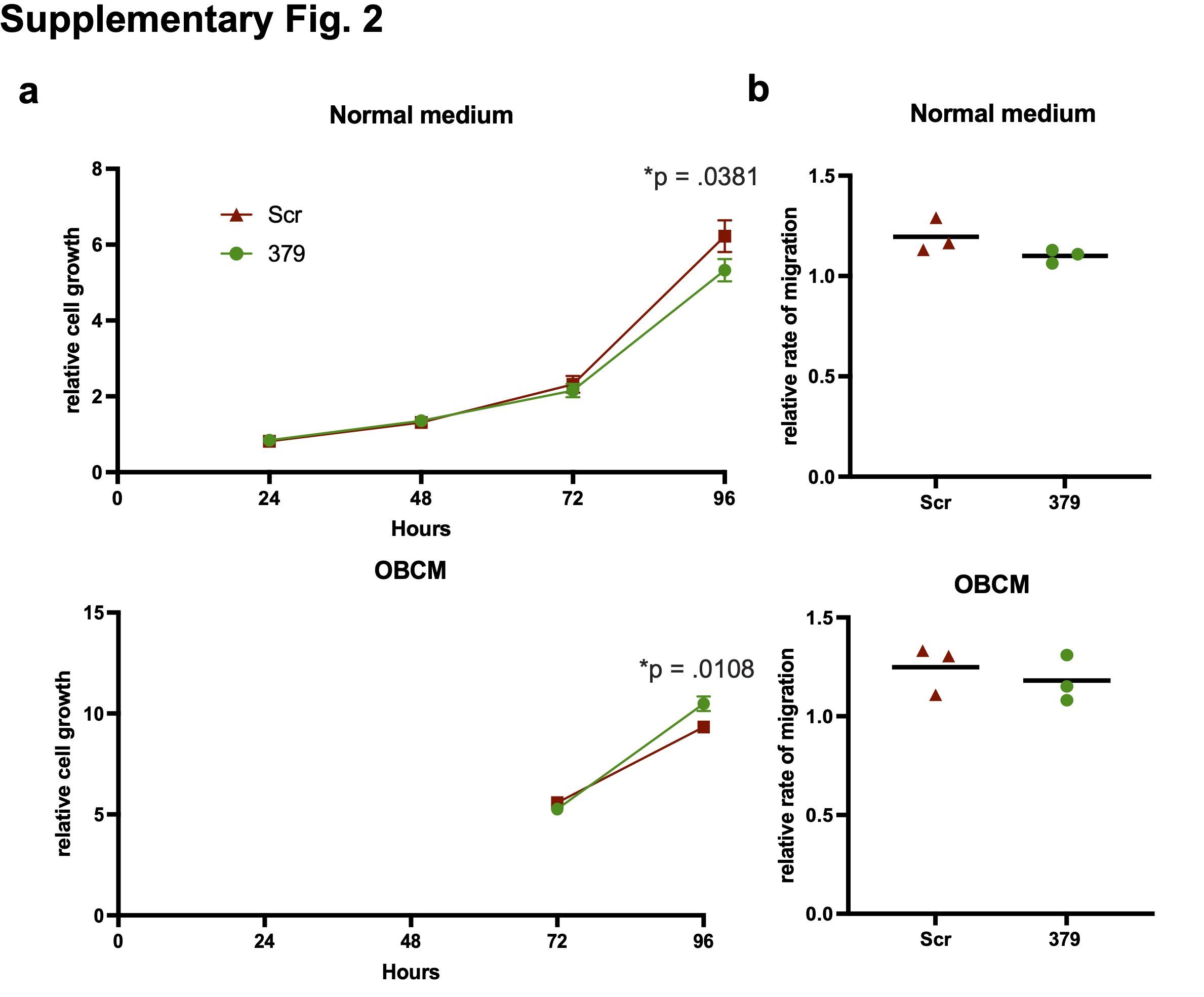

Supplement: Supplementary Figure 2 — Effect of miR-379 overexpression on LNCaP-ARhi cells in vitro. LNCaP-ARhi cells transduced with miR-379 were assessed for the effect of miR-379 on cell growth (A) and migration (B). For the SRB assays (A), the red boxes denote Scr control cells, and the green circles denote miR-379 overexpressed cells. For the migration (B) experiments, the red triangles denote Scr control cells, and the green circles denote miR-379 overexpressed cells. The experiments were performed in normal media and OBCM. Experiments were performed three times, and representative data is shown. Unpaired two-tailed Student’s t-tests were performed to compare the treatment groups to one another; *p < 0.05. Only statistically significant p values are shown in the figure. [file Image_2.tiff]

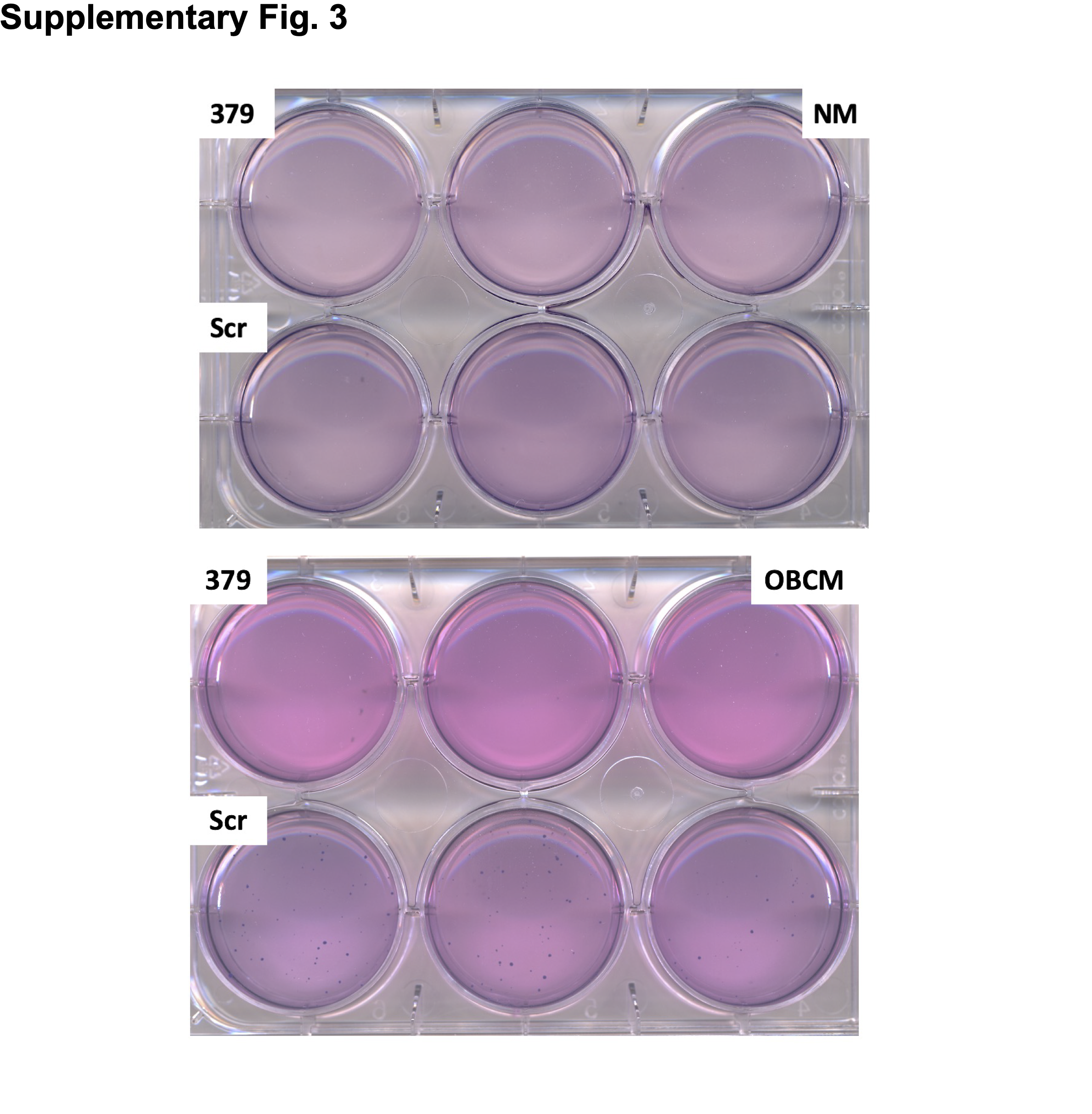

Supplement: Supplementary Figure 3 — Plates from the colony formation experiment. Scanned images of the 6-well plates from which the number of visible colonies was counted. The number of colonies can be seen in . [file Image_3.tiff]

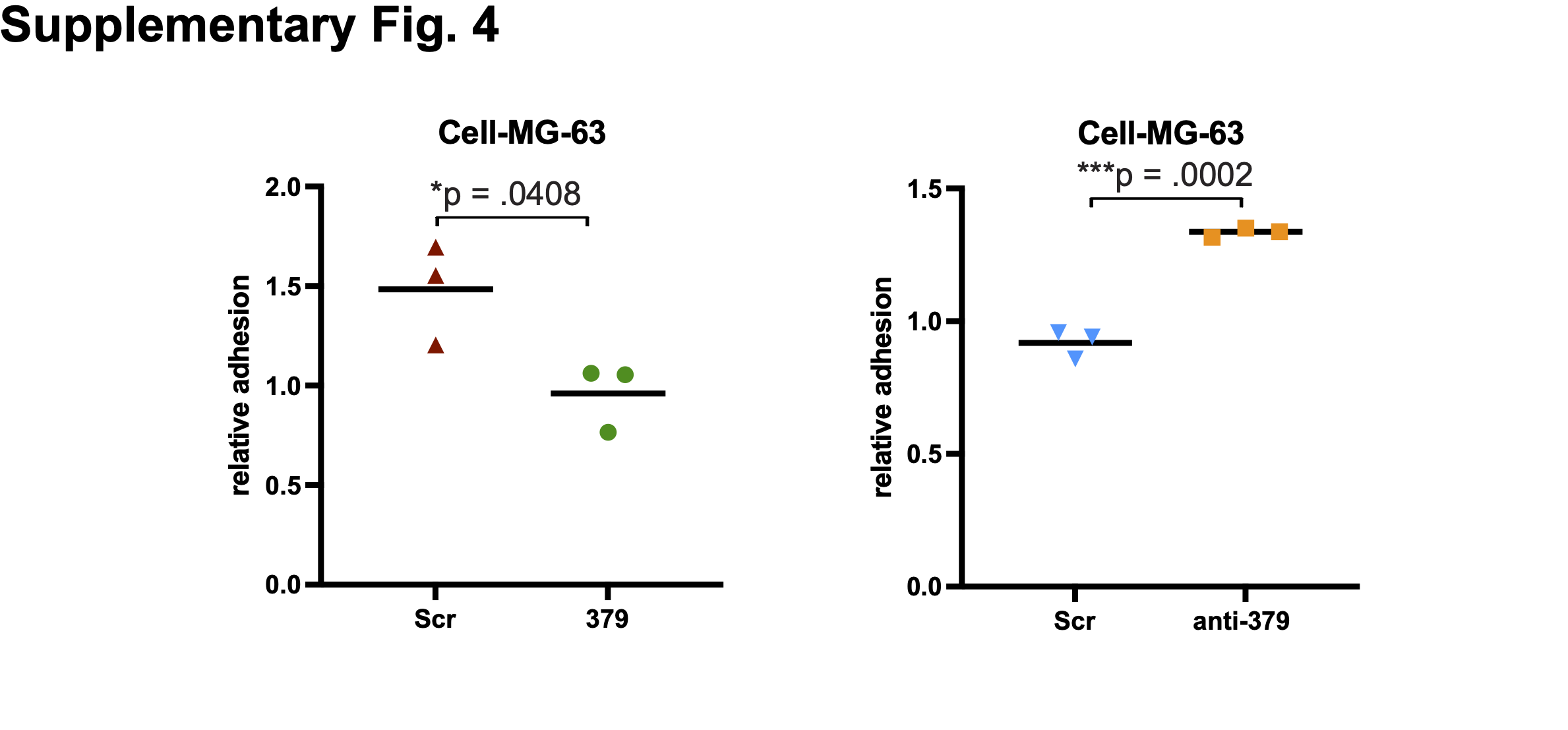

Supplement: Supplementary Figure 4 — Effect of miR-379 overexpression and downregulation on cellular adhesion to MG-63 cells. The red/blue triangles denote Scr control cells, and the green circles/orange boxes denote miR-379 and anti-miR-379 cells. Experiments were performed three times, and representative data is shown. Individual values of biological triplicates are shown as blue triangles and orange squares. Unpaired two-tailed Student’s t-tests were performed to compare the treatment groups to one another; *p < 0.05, ***p < 0.001. Only statistically significant p values are shown in the figure. [file Image_4.tiff]

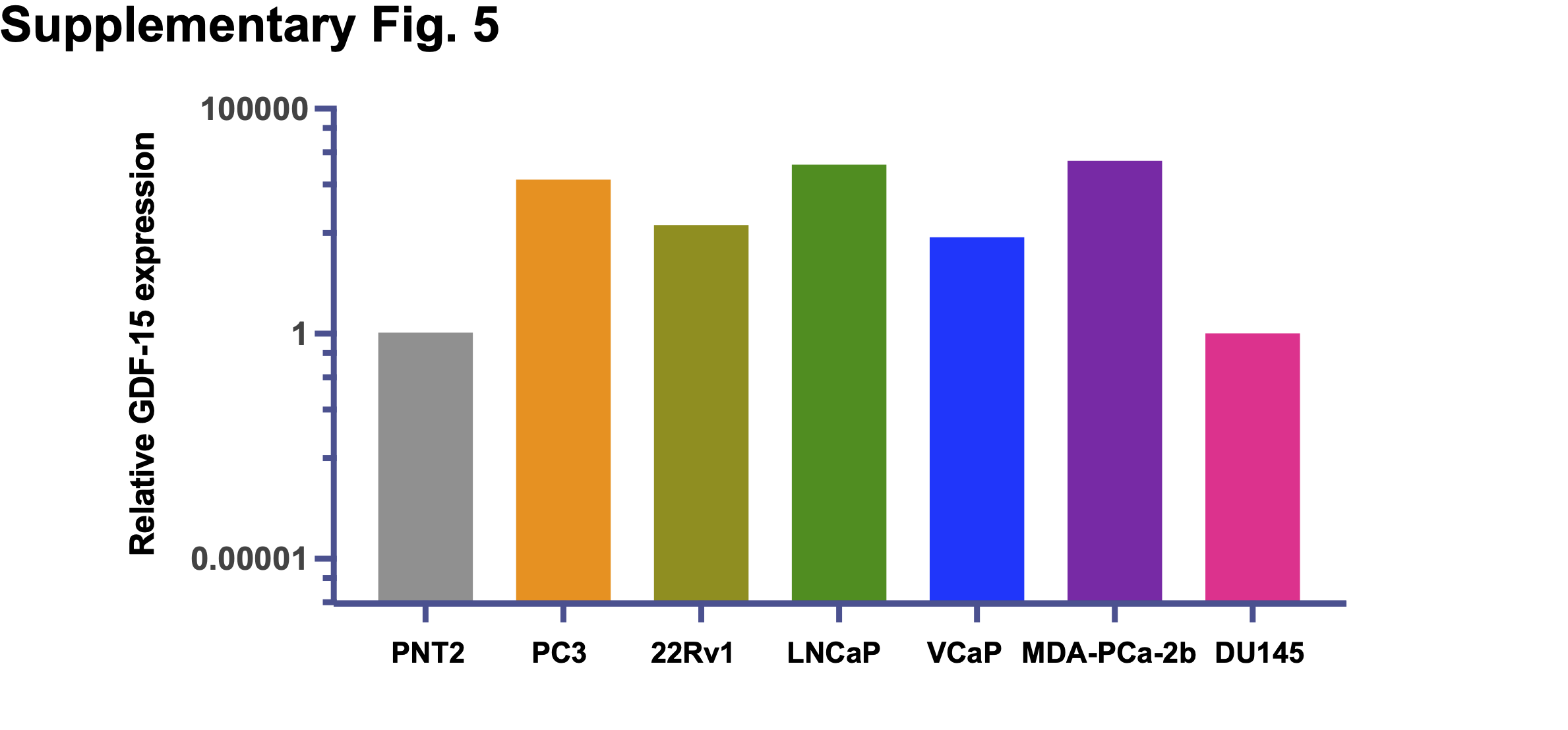

Supplement: Supplementary Figure 5 — GDF-15 expression levels in cell lines. The bar chart shows baseline expression of several prostate cancer cell lines and an immortalised normal prostate cell line (PNT2, grey). [file Image_5.tiff]
